# Comparative Secretome Profiling of *Arabidopsis* Seedlings Under Pi Starvation Unveils the Regulatory Role of PHR1

**DOI:** 10.3390/plants15182826

**Published:** 2026-09-15

**Authors:** Ran Xu, Jizhen Xie, Xueqing Duan, Piao Liu, Xingyi Zheng, Xiaoyang Li, Sheng Jiang, Wenjun Wang, Xixi Cai, Shengchao Ge, Yunfeng Liu, Yuan Su

**Affiliations:** 1 Guangxi Sugarcane Bio-Breeding Laboratory, State Key Laboratory for Conservation and Utilization of Subtropical Agro-Bioresources, College of Life Science and Technology, Guangxi University, Nanning 530004, China; 15194464495@163.com (R.X.); xjz1467113818@163.com (J.X.); lampduan@gmail.com (X.D.); 19850152315@163.com (P.L.); zhengxingyi@st.gxu.edu.cn (X.Z.); lixiaoy2018@126.com (X.L.); yaeaponia0722@163.com (S.J.); caixixi@gxu.edu.cn (X.C.); shengchaoge@gxu.edu.cn (S.G.); 2 Cloud-Clone Co., Ltd., Wuhan 430056, China; wangwenjun008@163.com; 3 Guangxi Technology Innovation Center for Microbial Resources Development and Utilization, Nanning 530004, China

**Keywords:** *Arabidopsis thaliana*, phosphate starvation, PHR1, secretome, phosphatase, redox metabolism, plant immunity

## Abstract

Phosphate (Pi) starvation severely limits plant growth, and the transcription factor PHR1 is a central regulator of the phosphate starvation response (PSR). However, its role in modulating the secreted proteome remains poorly understood. Here, we performed comparative secretome profiling of wild-type (WT) and *phr1* mutant *Arabidopsis* seedlings under Pi-sufficient (+P) and Pi-deficient (−P) conditions using LC–MS/MS. We quantified 2077 proteins from culture media across four biological replicates. Pi starvation induced 394 differentially abundant secreted proteins in WT, while the *phr1* mutation severely attenuated this response, particularly down-regulating 213 proteins under –P conditions. PHR1 was essential for the starvation-induced secretion of phosphate-acquisition enzymes, including GDPD6, PAP1, and RNS1, and its role in the promotion of extracellular phosphatases was confirmed by acid phosphatase activity assays. Additionally, PHR1 modulated redox-related and defense-related secreted proteins, linking nutrient stress with oxidative and immune signaling. Integrative transcriptomic analysis revealed partial concordance between protein and mRNA levels, suggesting both transcriptional and post-transcriptional regulation. Collectively, our findings establish PHR1 as a vital regulator of the Pi-starvation-induced secretome and provide new insights into plant adaptation to phosphorus limitation through coordinated protein secretion.

## 1. Introduction

Phosphorus (P) is an essential macronutrient that plays a central role as a constituent of key biomolecules, including nucleic acids, phospholipids, and ATP, and is thus indispensable for plant growth, metabolism, and development. However, the bioavailability of inorganic phosphate (Pi), the primary form of phosphorus assimilated by plants, is severely limited in most soils due to its rapid fixation into organic compounds or insoluble complexes with metal cations [1]. Consequently, plants frequently encounter Pi starvation, which imposes a major constraint on agricultural productivity and natural ecosystem dynamics. To cope with low Pi availability, plants have evolved a suite of sophisticated adaptive strategies, collectively termed the phosphate starvation response (PSR). These responses encompass morphological modifications such as the inhibition of primary root growth and the proliferation of lateral roots and root hairs, as well as biochemical and metabolic adjustments, including the enhanced secretion of acid phosphatases and organic acids, and the remobilization of internal Pi reserves [1,2,3].

At the molecular level, the PSR is orchestrated by a complex transcriptional regulatory network. Central to this network is the MYB-CC transcription factor PHOSPHATE STARVATION RESPONSE 1 (PHR1), which functions as a master regulator of Pi homeostasis in *Arabidopsis thaliana* [4,5]. Under Pi-sufficient conditions, PHR1 activity is repressed through its interaction with SPX domain-containing proteins, which act as sensors of cellular Pi status. When Pi becomes limiting, PHR1 is released and binds to a conserved cis-element, the PHR1-binding sequence (P1BS, GnATATnC), in the promoters of its target genes, thereby activating the expression of hundreds of genes involved in Pi acquisition, translocation, and recycling [6,7]. The pivotal role of PHR1 is underscored by the pleiotropic phenotypes of *phr1* mutants, which exhibit impaired PSR, reduced anthocyanin accumulation, and altered sugar and starch metabolism under Pi starvation [5,8].

While the transcriptional regulation of the PSR has been extensively studied, the downstream physiological and metabolic consequences of these transcriptional changes, particularly at the protein level, remain less well understood. Secreted proteins, including acid phosphatases, ribonucleases, and other enzymes involved in the mineralization of organic phosphorus in the rhizosphere, play a direct and critical role in enhancing Pi bioavailability and are key components of the plant’s adaptive response to Pi deficiency [2,9]. Despite their importance, a comprehensive analysis of the secretome, the complement of proteins secreted into the extracellular space, and its regulation by PHR1 during Pi starvation is lacking. Recent evidence has further highlighted the intricate interplay between PSR and other cellular processes, including plant immunity and carbon metabolism [7,10,11], suggesting that the secretory pathway may serve as a crucial nexus integrating these responses. For instance, PHR1 has been shown to modulate the root microbiome by suppressing plant immunity via the RALF-FERONIA signaling pathway, a process that likely involves the secretion of specific proteins [12,13].

To address these knowledge gaps, we employed a comparative secretome profiling approach to characterize the protein repertoire secreted by *Arabidopsis* seedlings under Pi-sufficient and Pi-deficient conditions. By comparing the secretome profiles of wild-type (WT) plants with those of the *phr1* mutant, we aimed to dissect the regulatory role of PHR1 in modulating the secreted proteome during Pi starvation. We hypothesized that PHR1 not only controls the expression of intracellular genes but also orchestrates a specific secreted protein response that is critical for external Pi mobilization and the coordination of plant–microbe interactions. Through this integrated approach, we identify novel PHR1-dependent secreted proteins and provide new insights into the molecular mechanisms by which plants adapt to phosphorus-limited environments.

## 2. Results

### 2.1. Identification of Proteins Secreted by WT and phr1 Seedlings

**Figure 1:**
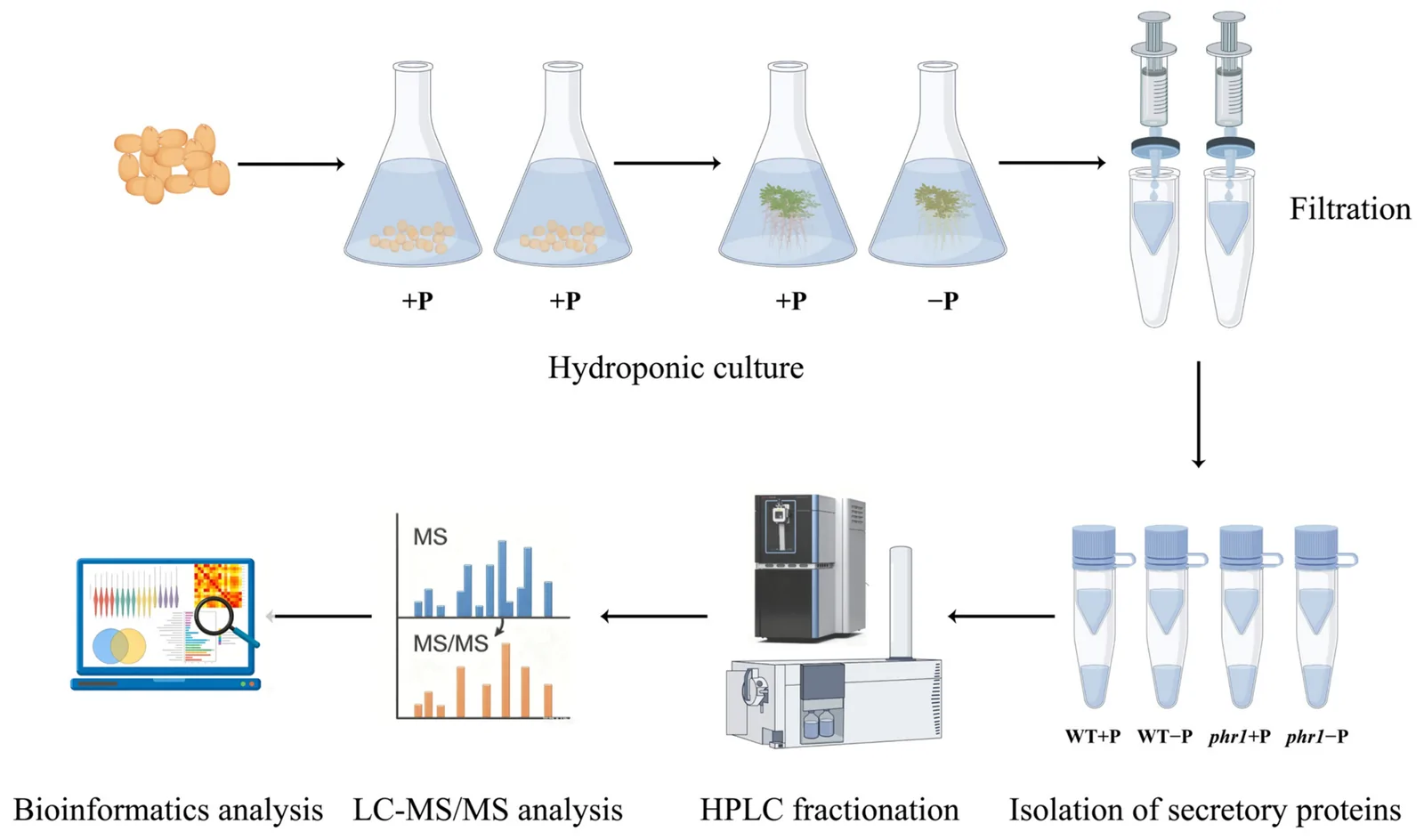
Experimental schematic workflow of *Arabidopsis* secretome analysis.

To collect secretome samples of *Arabidopsis* seedlings, we cultivated WT and *phr1* seedlings in an aseptic and hydroponic media system and harvested secreted and freely soluble proteins in Pi-sufficient (1 mM Pi, +P) media or starvation (0 mM Pi, −P) media (Figure 1). As expected, Pi limitation stress repressed the growth of *Arabidopsis* seedlings, while the *phr1* mutant exhibited more stunted growth and accumulated less biomass than WT under −P conditions, as transcriptions of hundreds of PSR genes were inhibited in the *phr1* mutant, but not under +P conditions (Supplementary Figure S1). Following membrane filtration, the protein samples from culture media were concentrated. To evaluate the purity of secretome samples, we probed immunoblots of *Arabidopsis* cell extracts and concentrated secretome samples with antibodies against cytoplasmic marker actin and performed activity assays of intracellular enzyme malate dehydrogenase (MDH). The Western blot results showed that actin bands were only detected in the cell lysate but not in secreted protein fractions (Supplementary Figure S2a). The MDH activity assay further demonstrated that almost no MDH activities were found in secretome samples compared with total protein extracts (Supplementary Figure S2b). Together, the data indicated that secreted protein preparations were largely free of leaked protein contamination from damaged or dying cells. Subsequently, secretome samples were subjected to quantitative analysis using liquid chromatography–tandem mass spectrometry (LC–MS/MS). A total of 11,804 high-quality valid spectra were identified with a utilization rate of 96%. These spectra were searched against the *Arabidopsis* Araport11 database, leading to the identification of 2153 proteins, of which 2077 were successfully quantified (Supplementary Table S1). For each treatment group (WT+P, WT−P, *phr1*+P, and *phr1*−P), four biological replicates clustered together based on Pearson correlation coefficients, with all correlation values exceeding 0.81 (Supplementary Figure S3a). Principal component analysis (PCA) further demonstrated that the four biological replicates within each treatment group exhibited consistent clustering (Supplementary Figure S3b), indicating that the proteomic dataset was reliable and suitable for subsequent analysis.

The N-terminal signal peptide (SP) directs the transportation of targeting proteins to the extracellular space via the endoplasmic reticulum/Golgi pathway. SP prediction was performed for all quantified secretome proteins using SignalP-6.0 [14]. A total of 699 proteins (33.65%) were predicted to contain an SP (Supplementary Figure S4a, Supplementary Table S2), which is in line with previous reports that 40 to 70% of the proteins identified in secretome studies lack an SP [15]. Gene Ontology (GO) enrichment analysis was performed on the secretome proteins, and the top 10 most significantly enriched terms were selected for each of the three GO categories, biological process (BP), molecular function (MF), and cellular component (CC). Notably, BP terms were predominantly associated with metabolic and catabolic processes, while MF terms highlighted catalytic and binding activities, and CC terms included extracellular regions and cell periphery components (Supplementary Figure S4b), indicating that a wide range of proteins were detected in the extracellular space.

### 2.2. Comparative Secretome Profiling Reveals PHR1-Dependent Reprogramming Under Pi Starvation

**Figure 2:**
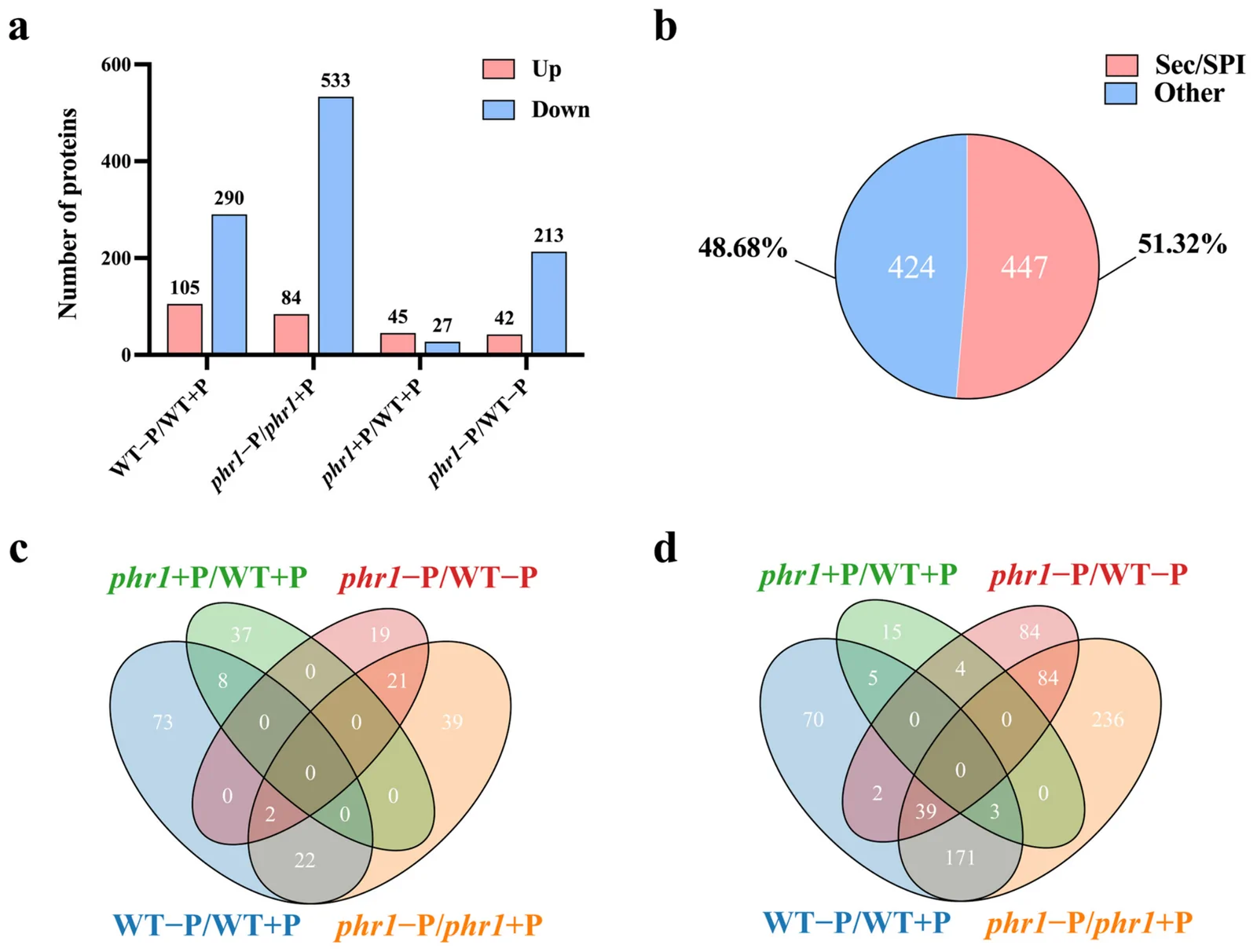
Analysis of differentially abundant proteins from the secretome profiling. (**a**) The number of up-regulated and down-regulated differentially abundant proteins in each comparison group. (**b**) N-terminal signal peptide prediction of all differentially abundant proteins by SignalP-6.0. (**c**) Venn diagram analysis of up-regulated differentially abundant proteins. (**d**) Venn diagram analysis of down-regulated differentially abundant proteins.

To investigate the molecular responses to Pi deficiency and the regulatory role of PHR1 in secreted proteins, we performed comparative proteomic analysis of secreted proteins from WT and *phr1* mutant seedlings grown under +P and −P conditions. A total of 871 differentially abundant proteins (DAPs) were identified across the four comparisons (WT−P/WT+P, *phr1*−P/*phr1*+P, *phr1*+P/WT+P, *phr1*−P/WT−P) with a cut-off of fold change (FC) > 1.3 (up-regulated) or <0.77 (down-regulated), *FDR* < 0.05, of which 447 (51.32%) proteins were predicted to contain an SP (Figure 2a,b, Supplementary Tables S2 and S3). In the WT−P/WT+P contrast, 105 proteins were up-regulated and 289 were down-regulated (Figure 2a), indicating a profound reprogramming of the secretome under Pi starvation. Notably, the number of secreted proteins regulated by Pi starvation differed markedly between WT and *phr1* mutant plants. Specifically, under +P conditions, 45 secreted proteins were significantly up-regulated and 27 were significantly down-regulated in the *phr1* mutant compared with WT. Under −P conditions, the number of up-regulated proteins in the *phr1* mutant remained similar (42 proteins), whereas the number of down-regulated proteins increased dramatically to 213 (Figure 2a), suggesting that the *phr1* mutation severely attenuates the Pi-starvation-induced secretory response. Venn diagram analysis further revealed that among the up-regulated DAPs, only 24 proteins were shared between WT and *phr1* in response to Pi deficiency, while the majority (81 proteins) were uniquely induced in the WT (Figure 2c). On the other hand, 77 down-regulated proteins were specific to the WT response, with 213 shared with *phr1* (Figure 2d). These results suggest that PHR1 plays a pivotal role in modulating the Pi-starvation-induced secretome.

### 2.3. GO and KEGG Functional Enrichment of Pi-Starvation-Responsive Secreted Proteins

**Figure 3:**
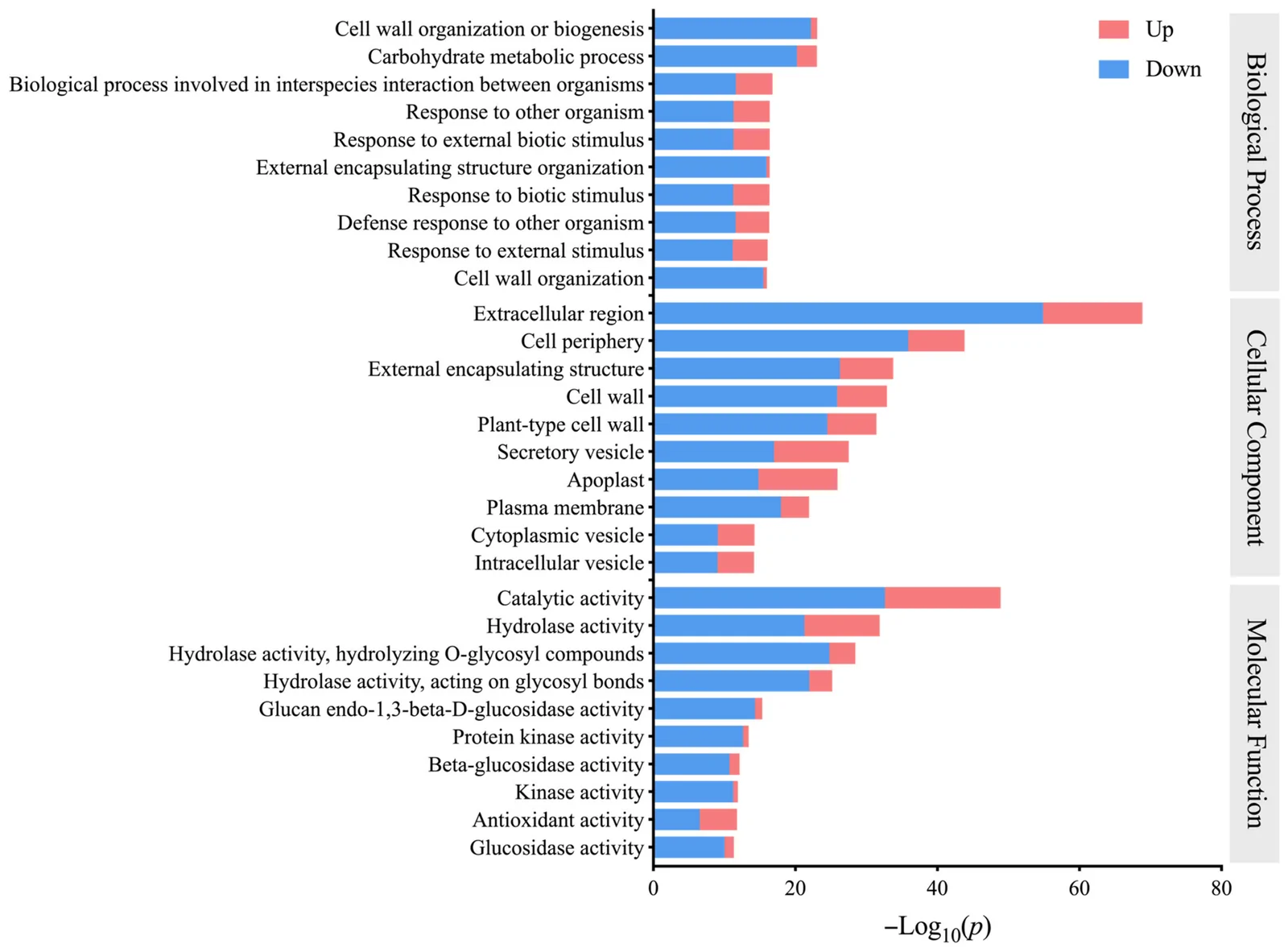
Gene Ontology (GO) functional enrichment of differentially abundant proteins in the WT−P/WT+P group. GO annotation is divided into three main categories: biological process (BP), cellular component (CC), and molecular function (MF). For each category, the top 10 most significantly enriched terms among differentially abundant proteins were selected. *p*-value < 0.05 represents significant functional enrichment.

To gain functional insights into the Pi-starvation-responsive proteins, we performed GO enrichment analysis on the DAPs identified in the WT−P/WT+P comparison. Among the top 10 enriched terms in each category, BP terms were predominantly associated with cell wall organization, stress responses, carbohydrate metabolism, and interspecies interaction, while MF terms highlighted catalytic and hydrolase activities. Notably, the extracellular region was identified as the most highly enriched term within the cellular component category, with plenty of proteins localized on the cell periphery and cell wall (Figure 3). These findings collectively indicate that Pi deficiency exerts a pronounced regulatory effect on the secreted proteome, likely involved in nutrient acquisition, stress response, intercellular signaling, and cell wall remodeling.

**Figure 4:**
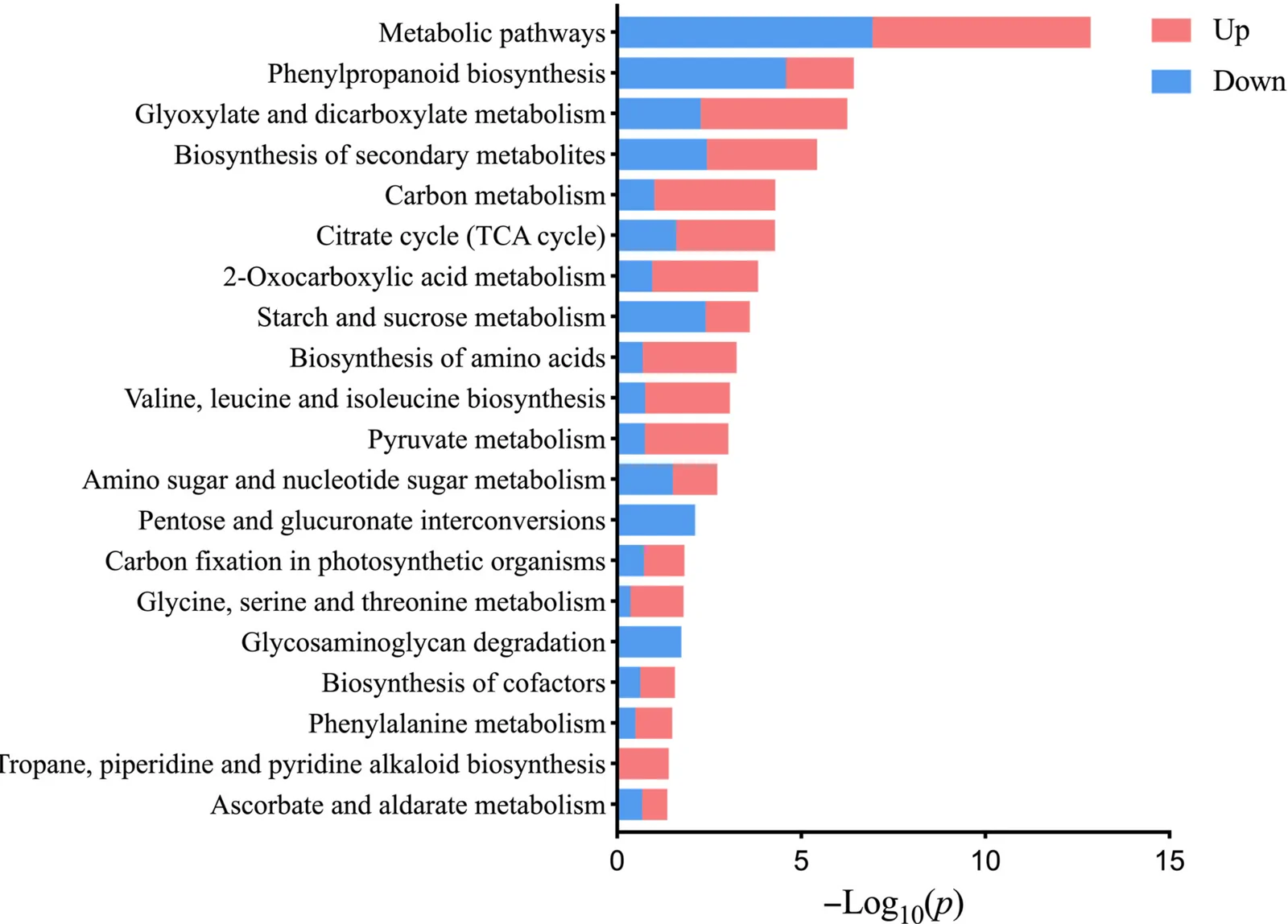
KEGG pathway enrichment for differentially abundant proteins in the WT−P/WT+P group. The top 20 pathways with the most significant enrichment are selected. *p*-value < 0.05 represents significant functional enrichment.

To further delineate the metabolic pathways affected by Pi starvation, we conducted KEGG pathway enrichment analysis on the same DAP set. The top 20 significantly enriched pathways included carbon metabolism, amino acid biosynthesis, and secondary metabolite synthesis, as well as pathways related to cofactor biosynthesis. Notably, within several of these pathways, the proportion of up-regulated versus down-regulated proteins varied substantially, with some pathways showing a clear bias toward up-regulation (e.g., carbohydrate metabolism) and others exhibiting a more balanced distribution (Figure 4). Collectively, these functional annotations suggest that Pi starvation may reprogram the secretome toward enhanced resource mobilization, stress adaptation, and intercellular communication.

### 2.4. PHR1-Dependent Regulation of Secreted Phosphatases and Redox Metabolism

**Figure 5:**
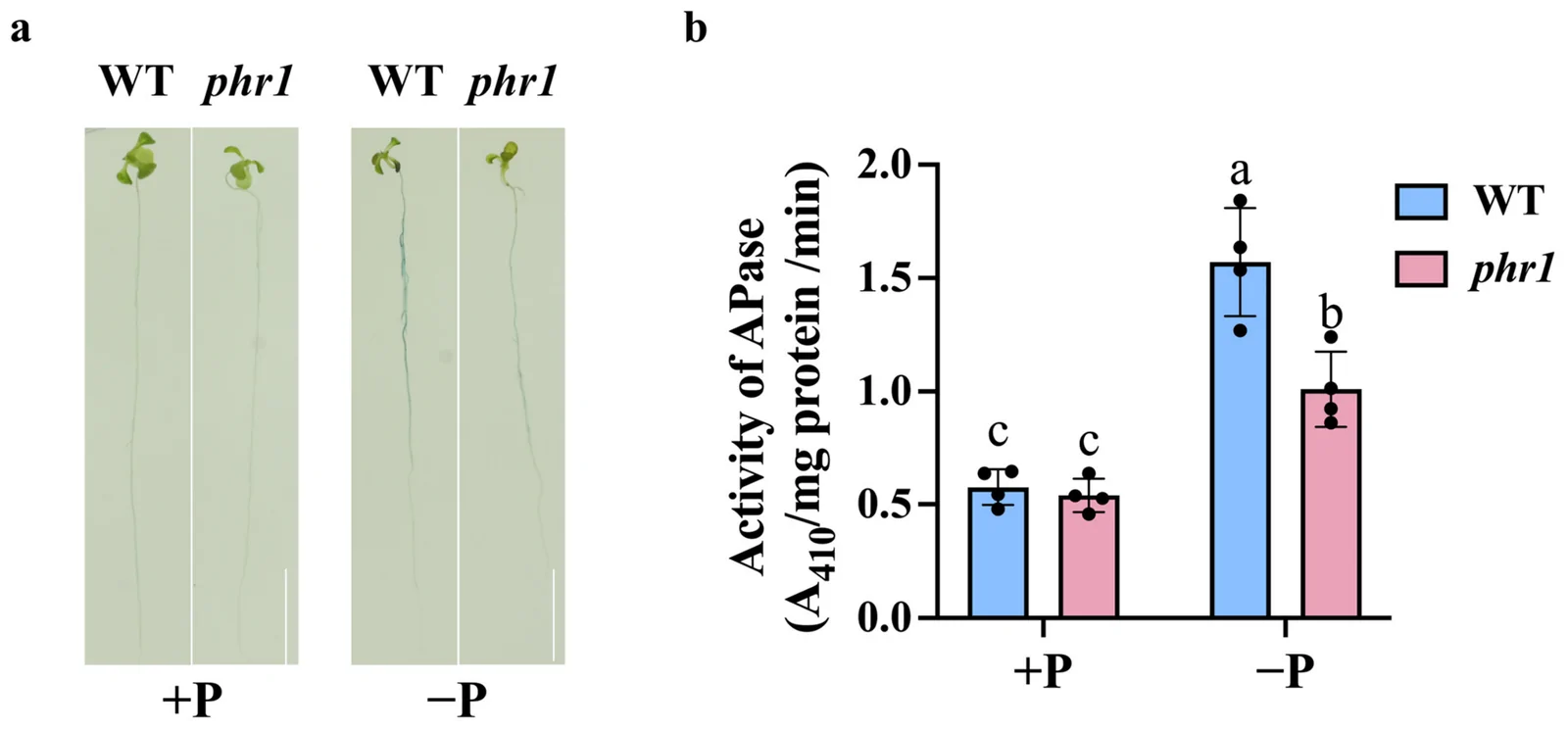
Acid phosphatase activity of *Arabidopsis* root surface and culture media. (**a**) Acid phosphatase staining of *Arabidopsis* root surface under +P or −P conditions. Scale bar = 1 cm. (**b**) Measurement of acid phosphatase activity of proteins isolated from the hydroponic +P or −P culture media. Data are means ± SD of four biological replicates. Different letters indicate significant differences using two-way ANOVA with Tukey’s HSD test (*p* < 0.05).

To identify key functional modules under PHR1 control, we examined secreted proteins involved in Pi acquisition and stress responses in the WT−P/WT+P dataset. Acid phosphatases, phosphodiesterases and nucleases are known to be secreted into the rhizosphere to utilize organic phosphorus under Pi deficiency. Consistent with previous reports, several secreted phosphatases were markedly up-regulated in WT upon Pi deprivation, including glycerophosphodiester phosphodiesterase 6 (GDPD6) [16], purple acid phosphatases (PAP1, PAP17) [17,18], and ribonuclease 1 (RNS1) [19], as well as 2-phosphoglycolate phosphatase 1 (PGLP1) and ATPases (RPT5A, ACA12), whose function in response to Pi stress has not been reported. For example, GDPD6 (AT5G08030) showed a 16.5-fold up-regulation in the WT−P/WT+P group, but this induction was severely compromised in the *phr1* mutant (only 1.2-fold in *phr1*−P/*phr1*+P), indicating a PHR1-dependent activation. Similar patterns were observed for PAP1, RNS1 and RPT5A, whose interaction *p*-value was 0.203, 0.041 and 0.032, respectively, by the genotype × phosphate interaction analyses (Table 1, Supplementary Figure S5). To assess extracellular acid phosphatase (APase) activities, histochemical staining revealed strong APase activity on the root surface of WT seedlings under −P conditions, whereas *phr1* roots exhibited markedly weaker staining (Figure 5a). Quantitative APase activity assays of culture media revealed that total secreted APase activity increased by more than 2.5-fold in WT under −P, while the *phr1* mutant showed a significantly blunted response (Figure 5b). These results provide direct evidence that PHR1 is essential for the Pi-starvation-induced secretion of functional phosphatases. In contrast, the accumulation of specific proteins, including PAP17 and PGLP1, was notably higher in *phr1* mutant seedlings relative to WT controls under Pi-limited conditions (Table 1), suggesting PHR1 is not necessary for their secretion induction.

**Figure 6:**
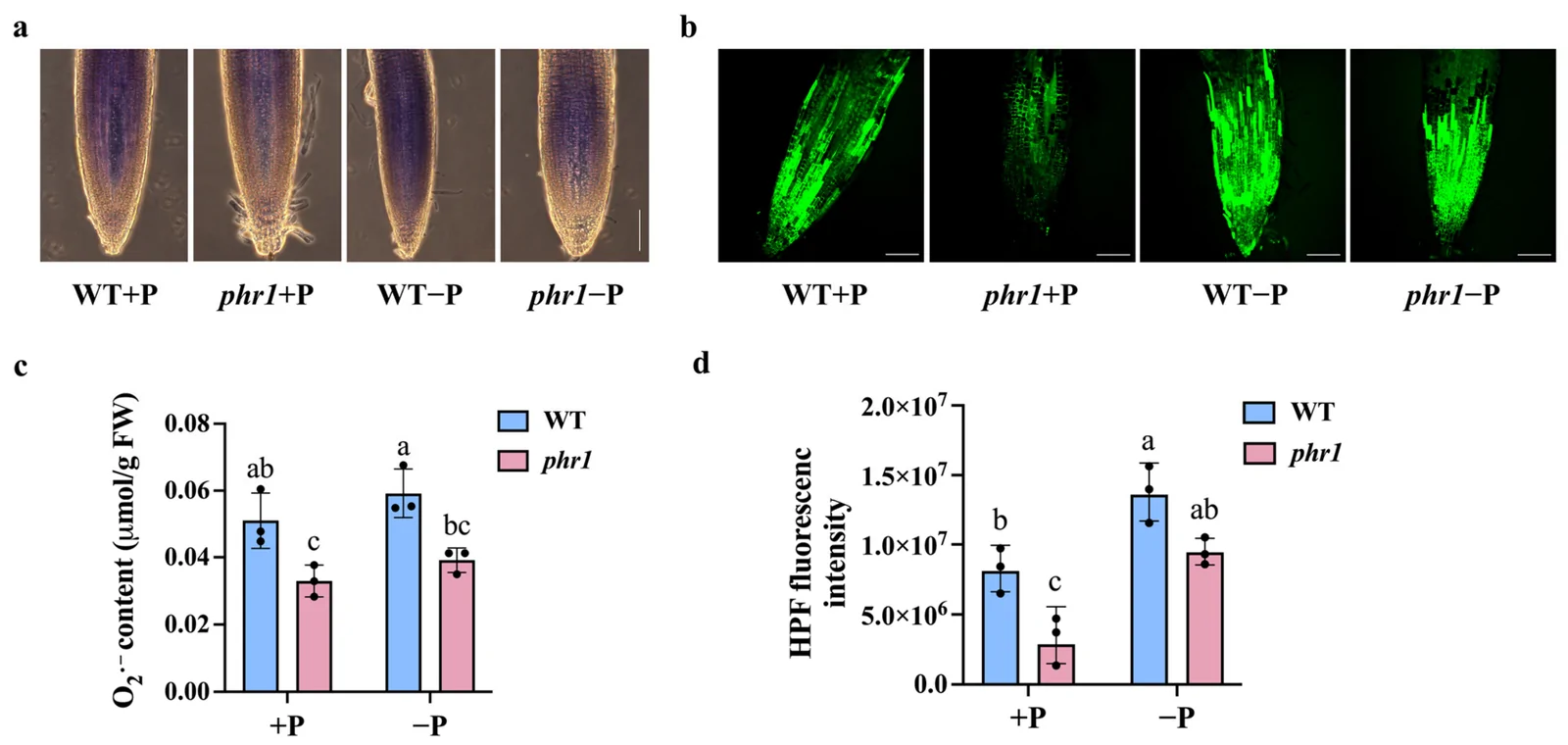
ROS content of WT and *phr1* plants either with or without Pi. (**a**) NBT staining of the root tips. Scale bar = 50 μm. (**b**) H_2_DCFDA staining of the root tips. Scale bar = 100 μm. (**c**) O_2_·^−^ content of the root tips. (**d**) Relative fluorescence intensity of H_2_DCFDA staining. Germinated seeds were grown in normal nutrient solution for 7 days and then transferred to solution either with or without Pi for another 2 days. Data are means ± SD of three biological replicates. Different letters indicate significant differences using two-way ANOVA with Tukey’s HSD test (*p* < 0.05).

In parallel, Pi starvation triggered a robust accumulation of redox metabolism-associated proteins. Peroxidases (PRX34, PRX52, AT3G01190), lipoxygenase 1 (LOX1), and oxidases (CUAO, GOX2) as well as redox regulators (CRK2, FAHD1A, GSTF9, GSH1, ARPN) were strongly induced in WT under −P conditions, of which the induction of LOX1, CRK2 and AT3G01190 was largely abolished or reversed in the *phr1* mutant, respectively (Table 2, Supplementary Figure S5), exhibiting secretion regulations by both PHR1-dependent and -independent pathways. Given that oxidases and peroxidases are involved in modulating the production or metabolism of reactive oxygen species (ROS), we assessed the intracellular ROS content in WT and *phr1* roots to compare with the extracellular situation. The O_2_·^−^ and H_2_O_2_ levels were visualized by NBT and H_2_DCFDA staining, respectively (Figure 6a,b). The O_2_·^−^ content was slightly elevated in WT and *phr1* mutant under −P condition compared with +P condition. However, the *phr1* mutant displayed significantly more reduced O_2_·^−^ levels than WT under both +P and −P conditions (Figure 6c), indicating that PHR1 seems to mediate O_2_·^−^ metabolism independently of Pi stress. By contrast, H_2_O_2_ levels in WT and *phr1* roots indicated by the relative H_2_DCFDA fluorescence intensity was promoted by Pi deprivation, while a significant difference was only observed under +P conditions between WT and *phr1* (Figure 6d). Collectively, the data suggested that the differential regulation patterns were likely applied to the intracellular ROS activities and those in the extracellular space. Overall, PHR1 coordinates both the secretory phosphatase system and the ROS signaling network to adapt to Pi deficiency to a varying extent.

### 2.5. PHR1-Mediated Pathogen Defense Responses

Further analysis of the DAPs revealed that numerous secreted proteins are involved in pathogen defense, including a few redox proteins identified in Table 2, suggesting a possible connection between phosphate starvation and plant defense. These proteins play their roles interacting with pathogens (fungi, bacteria, viruses) at various layers, including cell wall remodeling, ROS-based toxicity, antimicrobial enzyme secretion, and secondary metabolite production (Table 3). A subset of proteins, such as certain redox-related proteins, pathogenesis-related 4 (PR4), and calcium-dependent protein kinase 1 (CDPK1) were up-regulated in the WT−P/WT+P group, whereas their induction extents in the *phr1* mutant were relatively reduced (Table 3, Supplementary Figure S5), indicating PHR1 may be involved in the regulation of Pi-starvation-triggered plant immunity. However, experimental evidence is desired for future studies and verification.

### 2.6. Integrative Proteomic and Transcriptomic Analysis of PHR1-Dependent Regulation

**Figure 7:**
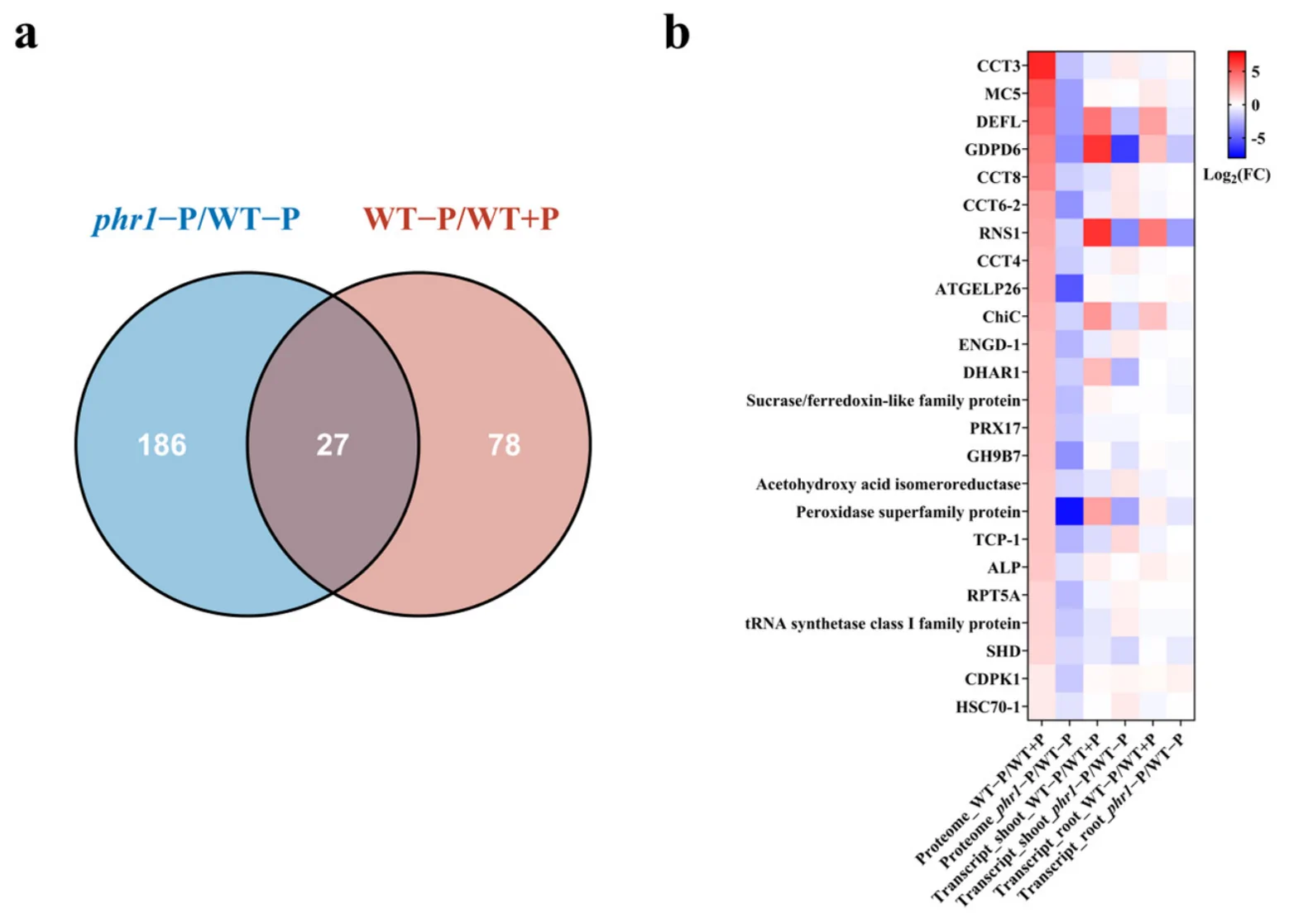
Integrated analysis of PHR1-dependent proteomics and transcriptomics under phosphate deficiency. (**a**) Venn diagram analysis of significantly down-regulated proteins in *phr1*-P/WT-P and up-regulated proteins in WT−P/WT+P. (**b**) Heatmap of significantly up-regulated proteins in the WT−P/WT+P group and down-regulated proteins in the *phr1*−P/WT−P group samples compared with those in the transcriptome of WT−P/WT+P and *phr1*−P/WT−P, respectively.

To evaluate the consistency between proteomic changes and transcriptional regulation, we integrated our secretome data with the corresponding transcriptomic profiles. We focused on the 105 up-regulated DAPs in the WT−P/WT+P group and matched them to the published transcriptome data [5], eventually yielding 97 hits (Figure S4, Supplementary Table S4). The results demonstrate that among 97 DAPs, 34 genes were significantly highly transcribed (FC ≥ 2) by Pi deprivation, while the transcript levels of 5 genes significantly declined (FC ≤ 0.5) in either shoots or roots, with 58 gene transcriptions not significantly changed. To further investigate the PHR1-dependent DAPs in the group, Venn diagram analysis was applied and the results show that 27 down-regulated proteins in the *phr1*−P/WT−P comparison overlapped with up-regulated proteins in the WT−P/WT+P comparison (Figure 7a). Heatmap analysis was subsequently conducted to project these 27 proteins to their transcription data and generated 24 hits (Supplementary Table S4). Interestingly, the results reveal that about 1/3 of proteins up-regulated in the WT−P/WT+P group exhibited concordant transcriptional up-regulation at the mRNA level in a PHR1-dependent manner in either shoots or roots (Figure 7b). In other words, the majority of PHR1-dependent DAPs did not share similar responding patterns in their transcript abundance to Pi stress. These results support a model in which PHR1 partially exerts its regulatory influence on the secretome through transcriptional activation of downstream target genes, although post-translational mechanisms may also contribute to a subset of DAPs.

## 3. Discussion

### 3.1. A Global Secretory Reprogramming Occurs Under Pi Starvation

It is well known that plants send bioactive molecules into the extracellular space (also called apoplast) to interact with pathogen effectors or modulate the extracellular environment [15]. Specifically, under Pi starvation, numerous chemical compounds such as organic acids and protons and enzymes such as APases and nucleases are released into the rhizosphere to better utilize fixed Pi and organic Pi, respectively [34]. Although secretome plays a critical role in external Pi mobilization and rhizosphere interactions, an up-to-date comprehensive secretome investigation has not been reported in *Arabidopsis*, especially under −P conditions. Although a few studies have explored the secretome of various plants (*Arabidopsis*, rice, rapeseed, pea and maize), either suspension cells (different from seedlings physiologically) were used in the study or the proteomic analysis was confined due to technique limitations at that time [9,34,35,36,37,38]. Furthermore, the adaptive strategies of plants to cope with Pi stress is centrally regulated by PHR1. While the transcriptional regulation of PSR has been extensively studied, the impact of PHR1 on the secreted proteome remains unclear. The present study provides a comprehensive secretome landscape of *Arabidopsis* seedlings responding to Pi deficiency and establishes PHR1 as a key regulator of this secretory response. The proteomic profiling identified 2077 secreted proteins in total, of which 699 proteins (33.65%) were predicted to contain a signal peptide (Figure S4a). A similar observation that majority of secreted proteins lack a canonical secretion signal has been reported [15]. Thus, unconventional protein secretion pathways such as extracellular vesicles may take effect and contribute the secretion process [39,40]. It is worth noting that although the secretome samples were revealed to be largely free of contamination by intracellular proteins by immunoblot detecting actin protein and MDH activity assays, we cannot exclude any leaked intracellular protein in the secretome due to the sensitivity of the above assays. Further, our results demonstrate that Pi starvation triggers a profound and widespread reprogramming of the secretome, with 395 differentially abundant secreted proteins identified in WT under −P versus +P conditions (Figure 2a). This extensive remodeling underscores the critical importance of secreted proteins in the plant’s adaptive response to phosphorus limitation. Notably, the *phr1* mutation dramatically impaired this secretory response, particularly in terms of protein down-regulation under Pi starvation, where 213 proteins were less abundant in the mutant compared with WT (Figure 2a). This observation is consistent with the established role of PHR1 as a master transcriptional activator of PSR genes [5,8] and extends its regulatory influence to the secreted proteome. Even though the specific function of PHR1 in response to Pi deficiency has been well studied, the application of the *phr1* complementation line (e.g., pPHR1::PHR1 in *phr1* background) would further clarify the role of PHR1 in secretome regulation. Anyhow, the finding that a substantial fraction of Pi-starvation-induced secreted proteins are dependent on PHR1 highlights the central position of this transcription factor in coordinating both intracellular and extracellular adaptive responses.

### 3.2. PHR1-Dependent Secretion of Phosphate-Acquisition Enzymes

Among the most striking PHR1-dependent responses were the induction of secreted phosphatases and phosphodiesterases, which are known to play a direct role in scavenging organic phosphorus from the rhizosphere [17,41,42]. Our proteomic data revealed that GDPD6, PAP1, PAP17, and RNS1 were strongly up-regulated in WT under Pi starvation, but this induction was largely abolished in the *phr1* mutant except for PAP17 (Table 1). GDPD6, a glycerophosphodiester phosphodiesterase, is involved in the hydrolysis of glycerophosphodiesters to release Pi [16], while purple acid phosphatases such as PAP17 are well-characterized secreted enzymes that dephosphorylate a broad range of organic phosphate substrates [17]. The PHR1-dependent regulation of these enzymes was further validated by histochemical staining and quantitative activity assays, which showed markedly reduced secreted APase activity in the *phr1* mutant under Pi deficiency. These results are in agreement with previous transcriptomic studies demonstrating that GDPD6, PAP1, and RNS1 are direct targets of PHR1 via the P1BS cis-element in their promoters [5]. Our findings provide proteomic evidence that PHR1 is indispensable for the functional secretion of most of these enzymes, reinforcing the notion that PHR1 controls not only the transcriptional activation but also the downstream execution of phosphate acquisition strategies. Interestingly, on the other hand, the induction of certain proteins such as PAP17 and PGLP1 was even more pronounced in the *phr1* mutant than in WT under Pi starvation, suggesting the existence of PHR1-independent pathways that may partially compensate for the loss of PHR1 function. This observation is consistent with reports that other transcription factors, including PHR1-like proteins (PHL1, PHL2) and WRKY family members, may act redundantly or in parallel to regulate specific aspects of the PSR [5,43,44].

### 3.3. Redox Metabolism and ROS Signaling: A PHR1-Mediated Link Between Nutrient Stress and Oxidative Balance

Pi starvation has been associated with alterations in cellular redox homeostasis and the accumulation of reactive oxygen species (ROS) in various plant species [45,46,47]. Our secretome analysis revealed that Pi deficiency triggered a robust accumulation of redox-related secreted proteins, including peroxidases (PRX34, PRX52, AT3G01190), lipoxygenase 1 (LOX1), and various oxidases and redox regulators (CRK2, FAHD1A, GSTF9) (Table 2). This observation suggests that Pi starvation may modulate the extracellular redox environment, potentially influencing both plant cell wall metabolism and rhizosphere interactions. Among these, the induction of CRK2 and AT3G01190 was largely dependent on PHR1, whereas others appeared to be regulated through PHR1-independent mechanisms. ROS staining experiments further revealed that *phr1* mutants exhibited significantly reduced intracellular O_2_·^−^ levels compared with WT under both +P and −P conditions, indicating that PHR1 may mediate O_2_·^−^ metabolism independently of Pi stress. The differential regulation patterns observed between O_2_·^−^ and H_2_O_2_ accumulation suggest that PHR1 exerts nuanced control over specific ROS species, possibly through the regulation of distinct sets of secretory oxidases and peroxidases. Given that extracellular ROS play pivotal roles in cell wall stiffening, pathogen defense, and root developmental signaling [48,49], our findings point to a model in which PHR1 may integrate Pi nutritional status with extracellular redox signaling to coordinate growth, defense, and stress adaptation. Future studies on these topics are desired to unveil detailed regulatory roles of PHR1.

### 3.4. PHR1 Modulates Plant Immunity: Convergence of Phosphate Starvation and Defense Signaling?

It was reported that phosphate starvation primes plant immunity [10]. However, whether secreted proteins or peptides are involved in the process is unclear. A notable finding of this study is the identification of numerous pathogen defense-related proteins induced by Pi starvation in PHR1-dependent and -independent means, providing an additional potential link between Pi stress and plant immunity and a possible regulatory role of PHR1. Recent reports demonstrate that Pi starvation and plant immunity are interconnected through the root microbiota and that PHR1 suppresses immunity to enable root colonization by beneficial microbes under low Pi conditions [10,11,12,13]. However, our data suggest a more nuanced scenario: while PHR1 may suppress certain immune pathways to promote mutualistic associations, it also appears to prime the secretion of specific defense-related proteins during Pi starvation. This dual role may reflect a fine-tuned balance between facilitating beneficial symbioses and maintaining basal immunity against pathogens. Further investigation into the functional significance of these secreted defense proteins under Pi-limiting conditions and their interplay with root-associated microbial communities will be an important direction for future research.

### 3.5. Discrepancy Between Transcriptome and Proteome: Insights into Post-Transcriptional Regulation

Integrative analysis of our proteomic data with publicly available transcriptomic datasets revealed that only approximately one-third of the PHR1-dependent secreted proteins exhibited concordant transcriptional up-regulation at the mRNA level. This partial concordance is consistent with previous studies reporting modest correlations between transcript and protein abundances in plants, particularly in stress responses [50,51,52]. Several factors may account for this discrepancy. First, the secretion process itself is subject to post-translational regulation, including signal peptide cleavage, glycosylation, and vesicular trafficking, which can modulate the abundance of secreted proteins independently of transcript levels [53,54]. Second, protein turnover rates and degradation pathways may differ substantially between individual secreted proteins, contributing to the decoupling of mRNA and protein dynamics. Third, the experimental differences between the proteomic and transcriptomic experiments, including tissues, developmental stages, growth conditions, and sampling times, may also contribute to the observed differences [55]. Nevertheless, the subset of proteins showing strong transcriptional and proteomic concordance such as GDPD6, PAP1, and RNS1 likely represents direct targets of PHR1-mediated transcriptional activation. The majority of discordant proteins, on the other hand, may be regulated through post-transcriptional mechanisms or may be indirect targets whose accumulation is influenced by downstream physiological changes triggered by PHR1 activity.

### 3.6. Biological Significance and Future Perspectives

The present study provides a comprehensive resource for understanding the PHR1-dependent regulation of the *Arabidopsis* secretome under Pi starvation. Our findings establish that PHR1 governs a multi-layered secretory response encompassing phosphate scavenging, redox modulation, and immune priming, thereby acting as a central coordinator of extracellular adaptation to phosphorus limitation. The identification of PHR1-independent secretory pathways suggests that additional regulatory mechanisms and transcription factors contribute to the fine-tuning of the Pi-starvation secretome, warranting further exploration. Moreover, the discovery of numerous secreted proteins with unknown functions among the DAPs highlights the need for functional characterization of these candidates in the context of Pi nutrition and plant–microbe interactions. Moving forward, it will be of great interest to investigate how the PHR1-regulated secretome influences root-microbiome interactions under natural soil conditions, as recent work has underscored the importance of root exudates and secreted proteins in shaping the rhizosphere microbiota [56,57,58,59]. Additionally, exploring the potential conservation of PHR1-mediated secretome regulation in crop species could offer valuable strategies for improving phosphorus-use efficiency in agricultural systems [3]. Collectively, our work advances the current understanding of plant adaptation to phosphorus deficiency and positions the secretome as a critical functional layer in the PHR1 signaling network.

## 4. Materials and Methods

### 4.1. Plant Materials and Growth Conditions

*Arabidopsis thaliana* plants used in this study are Columbia-0 (Col-0) ecotype. The T-DNA insertion mutant *phr1* (Salk_067629) was obtained from the *Arabidopsis* biological resource center (ABRC, https://abrc.osu.edu). The homozygous *phr1* mutant was confirmed by PCR-based genotyping using gene-specific primers (forward, 5′-GAGAGACCTCACACGCACTTC-3′; reverse, 5′-CTTTCTGGCGAACCTGTAGTG-3′) and the T-DNA left-border (LB, 5′-ATTTTGCCGATTTCGGAAC-3′) primer.

For Pi treatments in hydroponic culture, 300 seeds were used in each sample. Surface sterilization was performed by adding 1 mL of sodium hypochlorite solution containing 1.5% active chlorine, followed by 1 mL of 75% ethanol with vigorous vortexing for 3 min for each wash. Afterward, the seeds were rinsed 4–5 times with sterile deionized water (ddH_2_O). Finally, the seeds were placed at 4 °C in the dark for 48 h before being put into the growth media. The seeds were first cultured in liquid PNS medium supplemented with 1 mM Pi for 4–5 days, and then transferred into medium with 1 mM Pi or 0 mM Pi for another 9–10 days. The PNS medium comprises 1.5 mM KCl, 5 mM KNO_3_, 2 mM MgSO_4_, 2 mM Ca(NO_3_)_2_, 1 mM KH_2_PO_4_, 1× basal salt micronutrient solution (Coolaber, PM1961, Beijing, China), and 0.5% sucrose (Solarbio, S8271, Beijing, China), with the pH adjusted to 5.6–5.8. For the 0 mM Pi treatment, KH_2_PO_4_ was omitted from the PNS medium with supplement of 1.5 mM KCl. The seedlings were put in a growth chamber at 22 °C and under 16 h light (566 µmol/m^2^/s)/8 h dark photoperiod with 70 rpm shaking.

### 4.2. Collection and Concentration of Secreted Proteins from Hydroponic Medium

The hydroponic solution was collected and subjected to sterile filtration using a 33-mm-diameter Millex-GP 0.22 µm syringe filter (Sigma, SLGP033R, Darmstadt, Germany). The filtrate was kept on ice. To remove trace glycerol present in the ultrafiltration membrane, the Cobetter^®^ UF-15 3K centrifugal device (Cobetter, C615300) was rinsed prior to use. Subsequently, the filtrate was added to the centrifugal filter unit and centrifuged at 4000× *g* for 60 min at 4 °C until the volume was reduced to approximately 200 µL. The concentrate was transferred into a new centrifuge tube and this concentration step was repeated 3–4 times with the entire filtrate from each treatment. Four biological replicates were processed in parallel.

### 4.3. Protein Sample Preparation and In-Gel Digestion

The protein samples were quantified with a Bradford Protein Assay Kit (Beyotime, P006, Shanghai, China). Equal amounts of protein samples were subjected to SDS-PAGE electrophoresis. The gel pieces were washed with 800 µL of 50 mM NH_4_HCO_3_ containing 30% acetonitrile (ACN) with intermittent vortexing until the Coomassie blue stain was completely removed, after which the supernatant was discarded. The gel pieces were then immediately incubated with 800 µL of ddH_2_O for 10 min to terminate the reaction, followed by removal of the supernatant. Subsequently, the gel pieces were incubated with 800 µL of 50 mM NH_4_HCO_3_ for 20 min at room temperature, and the supernatant was discarded. For dehydration, 100 µL of 100% ACN was added and incubated for 5 min, after which the ACN was aspirated. This dehydration step was repeated until the gel pieces were completely shrunk and turned opaque white.

The dehydrated gel pieces were incubated with an appropriate volume of freshly prepared mixture containing tris (2-carboxyethyl) phosphine (TCEP) and chloroacetamide (CAA) dissolved in 50 mM NH_4_HCO_3_. The samples were incubated at 56 °C for 30 min in the dark to reduce disulfide bonds and alkylate free thiol groups. Following incubation, the supernatant was completely removed. The gel pieces were dehydrated again by adding 100 µL of 100% ACN for 5 min, which was subsequently aspirated; this step was repeated until the gel pieces were fully contracted, and residual ACN was removed by brief air-drying.

The dried gel pieces were rehydrated with an appropriate volume of trypsin solution (20 ng/µL in 50 mM NH_4_HCO_3_) and placed at 4 °C for approximately 30 min to allow complete swelling. After swelling, the excess trypsin solution was carefully removed, and an appropriate volume of 50 mM NH_4_HCO_3_ digestion buffer was added to cover the gel pieces. Overnight digestion was performed at 37 °C. Following digestion, the supernatant containing the digested peptides was transferred to a fresh microcentrifuge tube. The remaining gel pieces were subjected to sequential extraction with 100 µL of 60% ACN in 0.1% trifluoroacetic acid (TFA) under ice-cold bath sonication for 15 min. The supernatant from each extraction was collected and pooled with the previously obtained supernatant. This extraction procedure was repeated multiple times, and the combined peptide extracts were lyophilized to dryness. The dried peptide mixtures were desalted using C18 Stage Tips according to the manufacturer’s instructions, concentrated, and dried down. Finally, the peptide samples were reconstituted in 0.1% formic acid (FA) in water and stored for subsequent LC-MS/MS analysis.

### 4.4. DIA Mass Spectrometry Data Acquisition

200 ng of peptides from each sample was subjected to peptide separation and detection using a Vanquish Neo UHPLC system coupled to an Orbitrap Astral mass spectrometer (Thermo Fisher Scientific, Waltham, MA USA). The peptides were analyzed using a 180 samples per day (SPD) method with a 5 min gradient on the Neo UHPLC system. Briefly, the peptides were loaded onto a trap column (PepMap Neo 5 µm C18, 300 µm × 5 mm, Thermo Scientific) and then separated on an analytical column (µPAC Neo High Throughput column, Thermo Scientific, Waltham, MA USA) with a gradient of up to 45% ACN. Mass spectrometric analysis was performed using an EASY-Spray source in data-independent acquisition (DIA) mode. The electrospray voltage was set to 2.2 kV, and the detection was carried out in positive ion mode. The precursor ion scan range was set from 380 to 980 *m*/*z*. Full MS resolution was set to 240,000, with an AGC target of 500% and a maximum injection time of 3 ms. For MS/MS, the resolution was set to 80,000, with an AGC target of 500%, a maximum injection time of 3 ms, and an RF-lens of 40%. MS2 activation type was higher-energy collisional dissociation (HCD) with an isolation window of 2 Th, normalized collision energy of 25%, and a cycle time of 0.6 s.

### 4.5. DIA Data Search and Protein Identification

This study employed the DIA-NN software (v1.8.1) to perform combined retrieval and quantitative analysis of the raw DIA mass spectrometry data. The Araport11 database was used for mass spectrometry data retrieval. The core search parameters were set as follows: Trypsin was selected as digestion enzyme. A maximum of 1 missed cleavage site was defined for database search. Carbamidomethylation of cysteines was defined as fixed modification, while acetylation of protein N-terminal and oxidation of methionine was set as variable modifications for database searching. Maximum number of variable modifications was 1. Peptide length range was set from 7–30. Charge of peptide was from 1 to 4. The fragment ion *m*/*z* range was from 150–2000. The database search results were filtered and exported with <1% false discovery rate (FDR) at peptide-spectrum-matched level and protein level, respectively.

From the protein identification table, experimental data were filtered to retain only those proteins detected with non-empty/non-zero values in more than 50% of samples within any given group. The median value for each sample was computed, followed by the global median across all sample medians. A normalization factor for each sample was derived by dividing the global median by the sample-specific median, and normalization was performed by multiplying each sample’s data by its corresponding factor. Missing or zero values were imputed with half of the minimum non-zero value present in the data. Differential expression between groups was assessed using a two-tailed Student’s *t*-test, with multiple testing correction performed via the Benjamini–Hochberg procedure to calculate the false discovery rate (FDR). Proteins exhibiting a fold change (FC) > 1.3 or <0.77 and FDR < 0.05 were defined as significantly differentially abundant proteins.

### 4.6. Interaction Analysis of Genotype and Phosphate Treatment

Two-way ANOVA was performed using GraphPad Prism 11 to identify proteins affected by the interaction between genotype and phosphate treatment. Protein abundance (log2-transformed normalized intensity values) was used as the dependent variable, with genotype (WT and *phr1*) and phosphate treatment (+P and −P) as fixed factors in a statistical model that included main effects and their interaction. An interaction term (genotype × phosphate) with a *p*-value < 0.05 was considered statistically significant. The interaction plots were generated using the Matplotlib (v3.10.8) library in Python (v3.x), plotting the mean log2-transformed abundance for each protein across the WT and *phr1* genotypes under the +P and −P conditions. Error bars represent the standard deviation (SD) of four biological replicates under each condition.

### 4.7. Bioinformatics Analysis

Bioinformatics analyses were performed using R statistical computing software (stats package, version 4.4.2). Hierarchical clustering analysis and volcano plots were generated using the R language. For sequence annotation, information was retrieved from the UniProtKB/Swiss-Prot, Kyoto Encyclopedia of Genes and Genomes (KEGG), and Gene Ontology (GO) databases. GO and KEGG enrichment analyses were conducted using Fisher’s exact test with false discovery rate (FDR) correction for multiple comparisons. GO terms were classified into three categories: biological process (BP), molecular function (MF), and cellular component (CC). In Fisher’s exact test, GO terms and KEGG pathways with a *p* < 0.05 were considered to be statistically significant.

### 4.8. Immunoblotting

Concentrated proteins (8 μg) from hydroponic cultures and whole-cell lysates (2 μg) were resolved by 10% SDS-PAGE and electrotransferred onto PVDF membrane. The membrane was blocked with TBST buffer (20 mM Tris-HCl pH 7.5, 150 mM NaCl, 0.1% Tween-20) containing 5% (*w*/*v*) non-fat dry milk for 1 h at room temperature. Following blocking, the membrane was incubated with anti-Actin antibody (1:5000, Abbkine, ABL1050, Wuhan, China) for 1 h at room temperature. After three washes with TBST, the membrane was incubated with horseradish peroxidase (HRP)-conjugated secondary antibody (1:5000, Abmart, M21001, Shanghai, China) for 1 h at room temperature. Following three additional washes with TBST (10 min each), immunoreactive signals were detected using ECL chemiluminescent substrate.

### 4.9. Enzyme Activity Assay of MDH

MDH activity was measured using a commercial MDH activity assay kit (Solarbio, BC1040) followed by the manufacturer’s instructions. Briefly, the oxidation rate of the substrate NADH was monitored by the absorbance at 340 nm for 1 min at 37 °C. One unit of enzyme activity was defined as the amount of enzyme required to oxidize 1 nmol of NADH per minute per mg of protein under the assay conditions. Each sample was analyzed in three technical replicates. The experiment was performed twice with similar results.

### 4.10. Signal Peptide Prediction

The amino acid sequences of the target proteins were set in FASTA format. The protein sequences were submitted to the SignalP-6.0 web server (https://services.healthtech.dtu.dk/services/SignalP-6.0/, accessed on 5 September 2026) with the organism group set to Eukaryota. The predicted signal peptide type and corresponding probability values were obtained from the output.

### 4.11. Root-Associated Acid Phosphatase Staining

Root activity staining of acid phosphatase was performed as previously described [58]. *Arabidopsis* seeds were grown in PNS medium containing 1 mM Pi for 7 days and then transferred to PNS medium supplemented with either 1 mM Pi or 0 mM Pi for an additional 2 days. For staining, 0.5 g of agar was added to 100 mL of ddH_2_O and heated until completely dissolved. After the agar solution had cooled to approximately 55 °C, 1 mL of 100× BCIP stock solution (Coolaber, CT11281) was added and mixed thoroughly. The BCIP-containing agar solution was then evenly poured over the root of the seedlings and incubated at room temperature for 24 h.

### 4.12. Acid Phosphatase Activity Assay

Secreted APase activity was measured as described previously [60]. Secreted proteins (30 μL) enriched from the liquid culture medium were added to a reaction buffer pre-warmed to 37 °C (10 mM MgCl_2_, pH 4.9; 50 mM sodium acetate, stored at 4 °C). Then, 1.0 mg/mL p-nitrophenyl phosphate (pNPP) solution (Solarbio, IP0300) was rapidly added, and the mixture was incubated at 37 °C for 1 h. The reaction was terminated by adding 0.4 N NaOH solution with vigorous vortexing. The absorbance at 410 nm was measured using a microplate reader. Acid phosphatase activity was calculated as A_410_/mg protein/min.

### 4.13. Measurement of ROS Levels

The staining and quantification of reactive oxygen species (ROS) were carried out as previously described [34]. To detect H_2_O_2_ levels, root tips of WT and *phr1* plants were sampled and stained with 10 µM H_2_DCFDA (Sigma, 287810) for 30 min at room temperature. Fluorescence images were acquired using Olympus FV3000 laser confocal microscope (Tokyo, Japan) with excitation at 495 nm and emission at 515 nm. The green fluorescence signal intensity in root images was quantified using ImageJ software (1.54p), and the data were expressed as total fluorescence intensity per selected region of interest.

To visualize superoxide anion (O_2_·^−^) levels, roots were stained with nitroblue tetrazolium (NBT). Roots of hydroponically grown WT and *phr1* plants were stained using a Plant Tissue Reactive Oxygen Species Assay Kit (Coolaber, SK1816) following the manufacturer’s instructions. The working solution was prepared by thoroughly dissolving 100 mg NBT in 100 mL of diluent. After washing with distilled water, the roots were submerged in the NBT working solution and incubated at room temperature for 1.5–2 h in the dark. After incubation, the roots were rinsed 3–5 times with distilled water and subsequently stored in tissue preservation solution. All steps were carried out under light-protected conditions. The stained roots were observed and imaged under a light microscope.

To quantify O_2_·^−^ levels, hydroponically grown seedlings were rinsed 3–4 times with distilled water, and excess water on the root surfaces was quickly blotted dry. Root samples (0.1 g) were homogenized in extraction buffer at a ratio of 1 g tissue to 10 mL buffer (*w*/*v*) in an ice bath. After homogenization, the samples were centrifuged at 12,000 rpm for 10 min at 4 °C, and the supernatant was collected and kept on ice. The superoxide anion content was then determined using a Superoxide Anion Detection Kit (Solarbio, BC1290) according to the manufacturer’s protocol. The absorbance of the reaction mixture was measured at 530 nm, and the superoxide anion content (μmol/g fresh weight) was calculated based on the formula provided in the kit.

## 5. Conclusions

In this study, we performed a comparative quantitative secretome analysis of *Arabidopsis* WT and *phr1* mutant seedlings to dissect the regulatory role of PHR1 in the secreted protein response to Pi starvation. Our results demonstrate that Pi deficiency induces a global reprogramming of the secretome, and that PHR1 is a central regulator of this process. Specifically, the *phr1* mutation severely impaired the Pi-starvation-induced secretory response, particularly reducing the abundance of proteins normally up-regulated in WT. We established that PHR1 is essential for the functional secretion of key phosphate-acquisition enzymes, including GDPD6, PAP1, and RNS1, which are critical for external Pi scavenging. Furthermore, PHR1 extends its regulatory influence beyond nutrient acquisition to modulate redox-related and defense-related secreted proteins, positioning PHR1 as a potential hub that integrates Pi starvation with oxidative stress responses and plant immunity. Integrative transcriptomic analysis revealed that while a subset of PHR1-dependent secreted proteins are transcriptionally regulated, a substantial proportion exhibits discordant patterns between mRNA and protein abundance, implying that post-transcriptional and post-translational mechanisms also contribute to the PHR1-mediated regulation of the secretome.

Collectively, our findings establish the secretome as a critical functional layer in the PHR1 signaling network and highlight the multifaceted role of PHR1 in coordinating extracellular adaptation to phosphorus-limited environments. The identification of PHR1-independent secretory pathways further suggests that additional regulatory mechanisms operate in parallel to fine-tune the Pi-starvation secretome, warranting future investigation. Moreover, the functional characterization of the numerous secreted proteins of unknown function identified in this study, as well as their roles in root–microbiome interactions, represents a promising avenue for future research. Ultimately, understanding how the PHR1-regulated secretome functions under natural soil conditions may offer valuable strategies for enhancing phosphorus-use efficiency in crops, contributing to more sustainable agricultural practices.

## Figures and Tables

**Table 1:** Phosphate-starvation-induced secreted phosphatase.

Gene ID	WT−P/WT+P	FDR	*phr1*−P/*phr1*+P	FDR	*phr1*+P/WT+P	FDR	*phr1*−P/WT−P	FDR	Functional Annotation
AT5G08030	16.517	0.023	1.247	0.679	1.154	0.977	0.087	0.031	Glycerophosphodiester phosphodiesterase 6, GDPD6
AT3G17790	12.536	0.010	21.395	0.029	0.666	0.932	1.136	0.874	Purple acid phosphatase 17, PAP17
AT2G02990	7.087	0.005	2.433	0.045	1.100	0.981	0.378	0.024	Ribonuclease 1, RNS1
AT1G13750	4.662	0.038	2.516	0.344	1.380	0.040	0.745	0.780	Purple acid phosphatase 1, PAP1
AT5G36700	3.583	0.036	5.129	0.008	1.553	0.919	2.222	0.040	2-phosphoglycolate phosphatase 1, PGLP1
AT3G05530	2.576	0.030	0.444	0.273	1.232	0.929	0.212	0.212	Regulatory particle triple-A ATPase 5A, RPT5A
AT3G63380	1.408	0.043	1.707	0.019	0.809	0.919	0.980	0.937	Auto-inhibited Ca^2+^ ATPase 12, ACA12

**Table 2:** Redox metabolism-associated proteins.

Gene ID	WT−P/WT+P	FDR	*phr1*−P/*phr1*+P	FDR	*phr1*+P/WT+P	FDR	*phr1*−P/WT−P	FDR	Functional Annotation
AT3G49120	61.321	0.034	56.018	0.227	1.069	0.936	0.977	0.987	Peroxidase 34, PRX34
AT1G70520	29.902	0.041	1.403	0.733	14.146	0.919	0.664	0.757	Cysteine-rich RLK2, CRK2
AT1G55020	18.066	0.021	6.840	0.179	6.750	0.040	2.556	0.452	Lipoxygenase 1, LOX1
AT4G12290	13.106	0.013	15.993	0.197	1.937	0.919	2.364	0.515	Copper amine oxidase, CUAO
AT5G05340	9.924	0.012	26.998	0.022	0.627	0.919	1.704	0.490	Peroxidase 52, PRX52
AT4G15940	3.741	0.032	1.764	0.333	2.211	0.919	1.043	0.967	Fumarylacetoacetate hydrolase domain containing protein 1A, FAHD1A
AT3G14415	3.572	0.036	2.252	0.366	3.842	0.045	2.422	0.515	Glycolate oxidase 2, GOX2
AT3G01190	3.530	0.037	0.030	0.281	0.651	0.919	0.006	0.029	Peroxidase superfamily protein
AT2G30860	3.080	0.041	10.161	0.183	1.304	0.919	4.301	0.409	Glutathione S-transferase PHI 9, GSTF9
AT4G23100	2.805	0.044	3.803	0.242	1.296	0.920	1.758	0.597	Glutamate-cysteine ligase 1, GSH1
AT2G02850	2.790	0.013	5.068	0.027	0.751	0.919	1.365	0.589	Plantacyanin, ARPN
AT4G20830	2.532	0.029	6.656	0.048	0.750	0.924	1.970	0.489	Oligogalacturonide oxidase 1, OGOX1

**Table 3:** Pathogen-response-related proteins.

Gene ID	WT−P/WT+P	FDR	*phr1*−P/*phr1*+P	FDR	*phr1*+P/WT+P	FDR	*phr1*−P/WT−P	FDR	Functional annotation	Responsive Pathogen	References
AT3G49120	61.321	0.034	56.018	0.227	1.069	0.936	0.977	0.987	Peroxidase 34, PRX34	*Alternaria brassicicola*	[20]
AT1G70520	29.902	0.041	1.403	0.733	14.146	0.919	0.664	0.757	Cysteine-rich RLK2, CRK2	*Pseudomonas syringae* pv tomato DC3000	[21]
AT1G55020	18.066	0.021	6.840	0.179	6.750	0.040	2.556	0.452	Lipoxygenase 1, LOX1	*Xanthomonas campestris*	[22]
AT5G60270	9.301	0.012	8.156	0.313	0.664	0.959	0.582	0.594	L-type lectin receptor kinase I.7, LecRK-I.7	*Verticillium dahliae*	[23]
AT1G33590	8.708	0.009	6.627	0.207	1.885	0.934	1.434	0.711	Leucine-rich repeat (LRR) family protein	*Alternaria brassicicola*	[24]
AT4G16260	6.587	0.044	4.649	0.268	1.149	0.957	0.811	0.863	Putative beta-1,3-endoglucanase	*Alternaria brassicicola*	[25]
AT3G04720	6.383	0.050	2.957	0.281	1.258	0.926	0.583	0.620	Pathogenesis-related 4, PR4	*Botrytis cinerea*	[26]
AT5G06320	5.428	0.032	6.095	0.194	1.533	0.919	1.722	0.602	NDR1/HIN1-like 3, NHL3	*Pseudomonas syringae* pv tomato DC3000	[27]
AT3G14415	3.572	0.036	2.252	0.366	3.842	0.045	2.422	0.515	Glycolate oxidase 2, GOX2	*Erwinia amylovora*	[28]
AT2G43510	3.277	0.009	6.149	0.022	0.994	0.999	1.865	0.380	Trypsin inhibitor protein 1, TI1	*Cabbage leaf curl virus*	[29]
AT2G30860	3.080	0.041	10.161	0.183	1.304	0.919	4.301	0.409	Glutathione S-transferase PHI 9, GSTF9	*Verticillium dahliae*	[30]
AT4G23100	2.805	0.044	3.803	0.242	1.296	0.920	1.758	0.597	Glutamate-cysteine ligase 1, GSH1	*Colletotrichum*	[31]
AT4G20830	2.532	0.029	6.657	0.048	0.750	0.924	1.970	0.489	Oligogalacturonide oxidase 1, OGOX1	*Botrytis cinerea*	[32]
AT2G43570	2.216	0.023	5.996	0.005	1.330	0.919	3.599	0.024	Chitinase, CHI	*Verticillium dahliae*	[23]
AT3G22600	1.664	0.044	5.363	0.175	1.431	0.919	4.614	0.360	Glycosylphosphatidylinositol-anchored lipid protein transfer 5, LTPG5	*Pseudomonas syringae* pv tomato DC3000 and *Botrytis cinerea*	[33]
AT1G18890	1.613	0.044	1.234	0.790	0.398	0.045	0.305	0.031	Calcium-dependent protein kinase 1, CDPK1	*Cabbage leaf curl virus*	[29]

## Data Availability

The mass spectrometry proteomics raw data have been deposited to the ProteomeXchange consortium via the PRIDE [61] partner repository with the dataset identifier PXD081553.

## Supplementary Materials

The following supporting information can be downloaded at https://www.mdpi.com/article/10.3390/plants15182826/s1, Figure S1: Growth phenotypes of *Arabidopsis* seedlings in Pi sufficient and starvation media; Figure S2: Validation of sample repeatability; Figure S3: Functional analysis of all the secreted proteins of the secretome; Figure S4: Integrated analysis of up-regulated secretome and transcriptomics in WT−P/WT+P; Figure S5. Interaction analysis of genotype and phosphate treatment on proteins of tables 1-3. Figure S6. Integrated analyses of up-regulated secretome and transcriptomics in WT−P/WT+P. Table S1: All proteins identified and quantified in this study; Table S2: Signal peptide prediction; Table S3: Differentially abundant proteins of the secretome; Table S4: Integration of proteomic and transcriptomic data.

## Abbreviations

The following abbreviations are used in this manuscript:

PSR	Phosphate starvation response
PHR1	PHOSPHATE STARVATION RESPONSE 1
WT	Wild type
PCA	Principal component analysis
SP	Signal peptide
DAP	Differentially abundant protein
FC	Fold change
GO	Gene ontology
KEGG	Kyoto encyclopedia of genes and genomes
APase	Acid phosphatase
ROS	Reactive oxygen species
PNS	Plant nutrient solution
