# Supplementary material for: Comparative Secretome Profiling of Arabidopsis Seedlings Under Pi Starvation Unveils the Regulatory Role of PHR1
Source: Plants (Basel). 2026 Sep 15;15(18):2826. doi: 10.3390/plants15182826 (PMC13610808; doi:10.3390/plants15182826)
Supplement: Supplementary file 1 [file plants-15-02826-s001.zip › Supplementary figures.pdf]

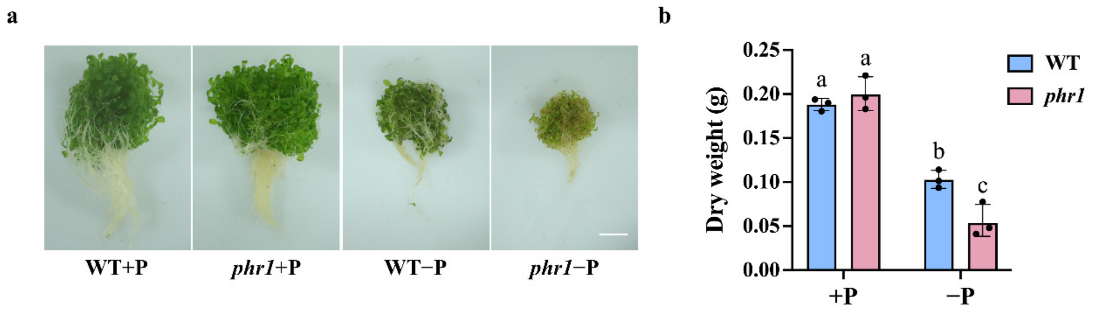

Supplementary Figure S1. Growth phenotypes of Arabidopsis seedlings in Pi sufficient and starvation media. (**a**) Phenotypes of WT and *phr1* seedlings after different Pi treatments. 6.0 mg of seeds were germinated and cultured in +Pi media for 4 days, followed by transferring into +Pi and −Pi medium for another 9 days. (**b**) Dry weight of seedlings in (a). Data are presented as means ± standard deviation (SD) of three independent biological replicates. Statistical significance was analyzed by two-way ANOVA analysis with Turkey test and represented by different letters ($p < 0.05$).

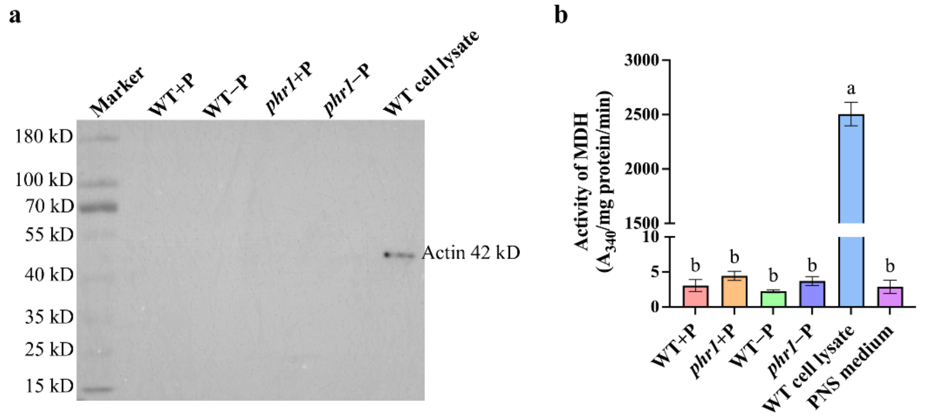

Supplementary Figure S2. Assessment of intracellular protein contamination in secreteome fractions from hydroponic cultures. **(a)** Immunoblot analysis was applied using anti-Actin antibody to secretome samples (8 μg each) and total cell lysate (2 μg) of WT Arabidopsis seedlings as a positive control. **(b)** Enzymatic activity assay of intracellular malate dehydrogenase (MDH) in the same protein fractions. The PNS culture medium was used as a negative control. Data are presented as mean ± SD (n = 3 technical replicates). Different lowercase letters indicate statistically significant differences among groups ($p < 0.05$, one-way ANOVA followed by Tukey's post-hoc test). This experiment was repeated twice with consistent results.

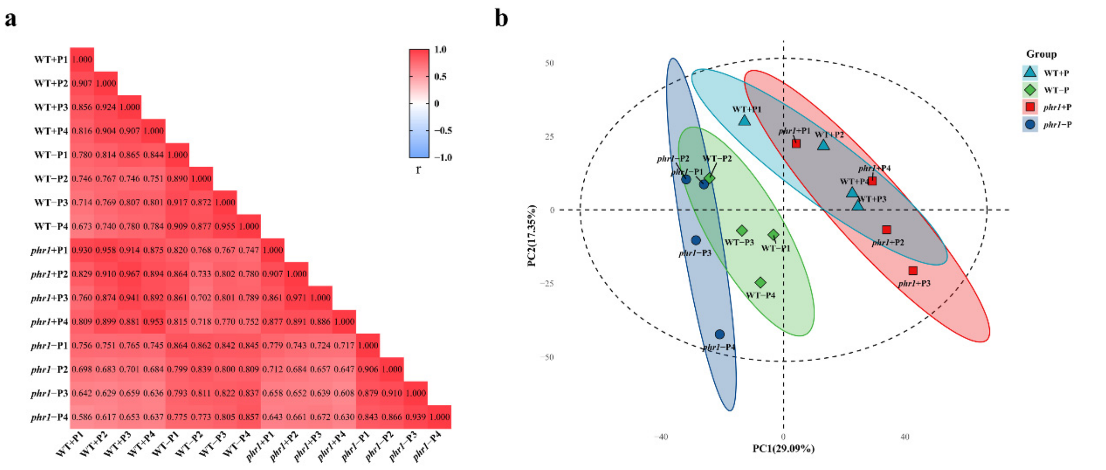

Supplementary Figure S3. Validation of sample repeatability. (**a**) Correlation heatmap of experimental samples. (**b**) Principal component analysis (PCA) analysis of experimental samples.

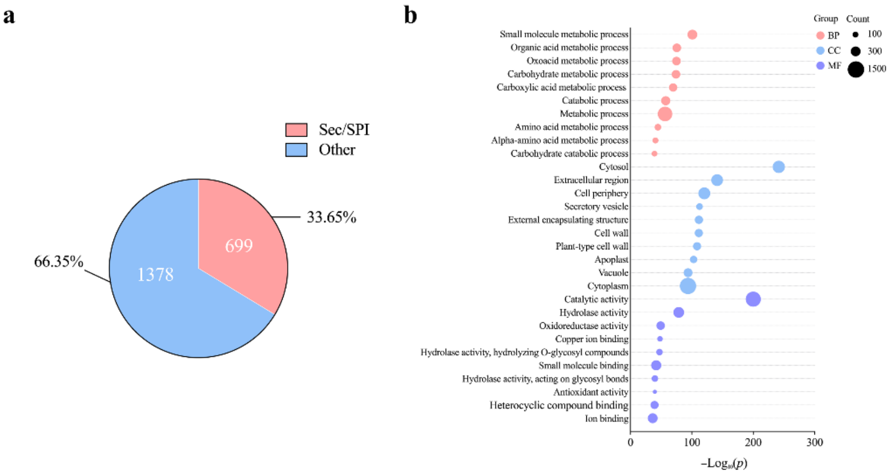

Supplementary Figure S4. Functional analysis of all the secreted proteins in the secretome. (**a**) Signal peptide prediction for the secretome proteins by SignalP-6.0. (**b**) GO functional enrichment of the secretome proteins. The top 10 most significantly enriched terms in each category (Biological Process, BP; Cellular Component, CC; and Molecular Function, MF) are presented. The *p*-value indicates the degree of enrichment, and values less than 0.05 represent significant functional enrichment.

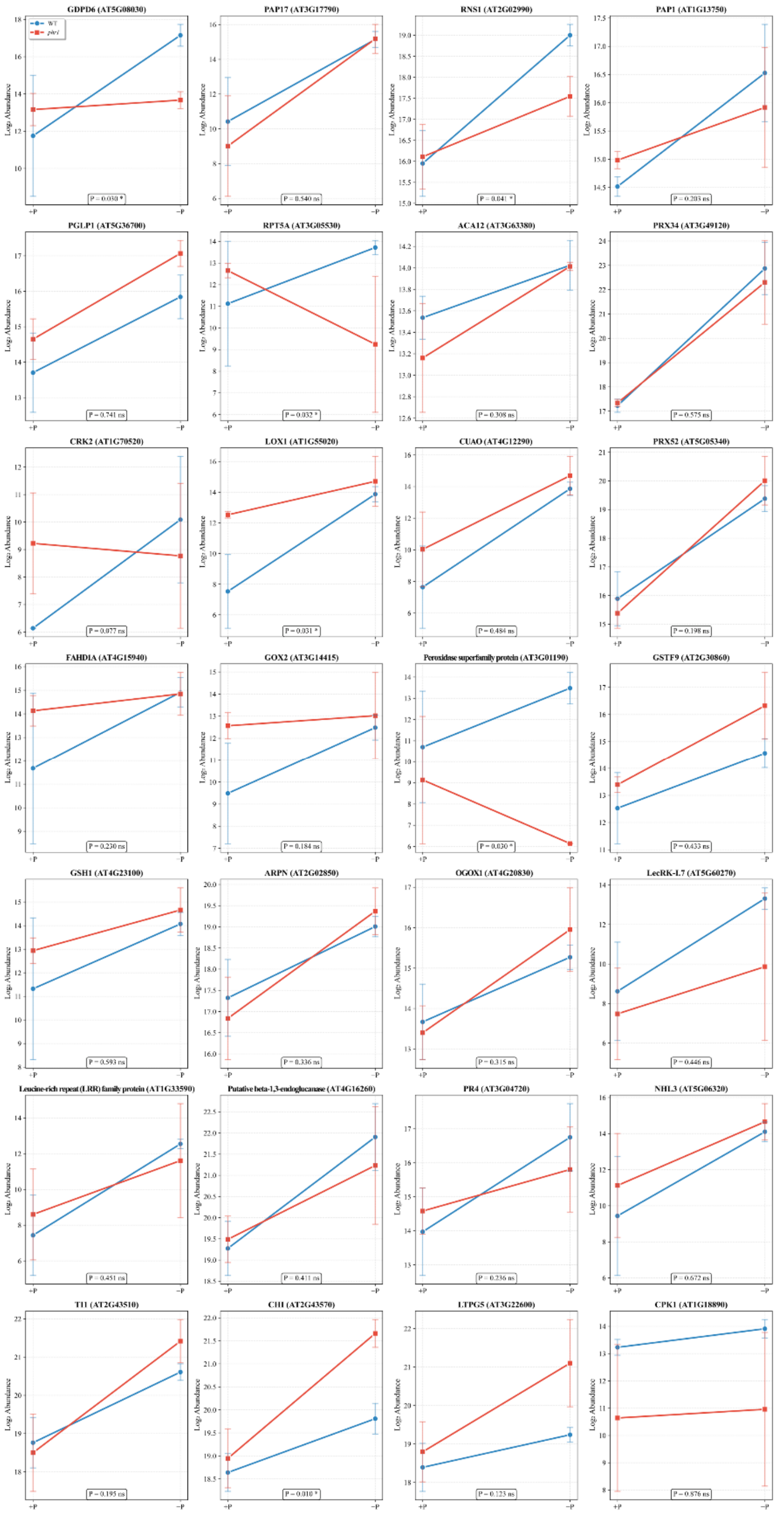

Supplementary Figure S5. Interaction analysis of genotype and phosphate treatment on proteins of tables 1-3. The genotype (WT and *phr1*) × phosphate (+P and −P) interaction was determined by two-way ANOVA analyses. Error bars indicate standard deviations (SD) from four biological replicates per treatment condition. The lines connecting the two phosphate treatments for the same genotype are used to visualize the direction and magnitude of phosphate responsiveness. The *p*-value for the interaction term is shown within each subpanel, with $p < 0.05$ considered statistically significant.

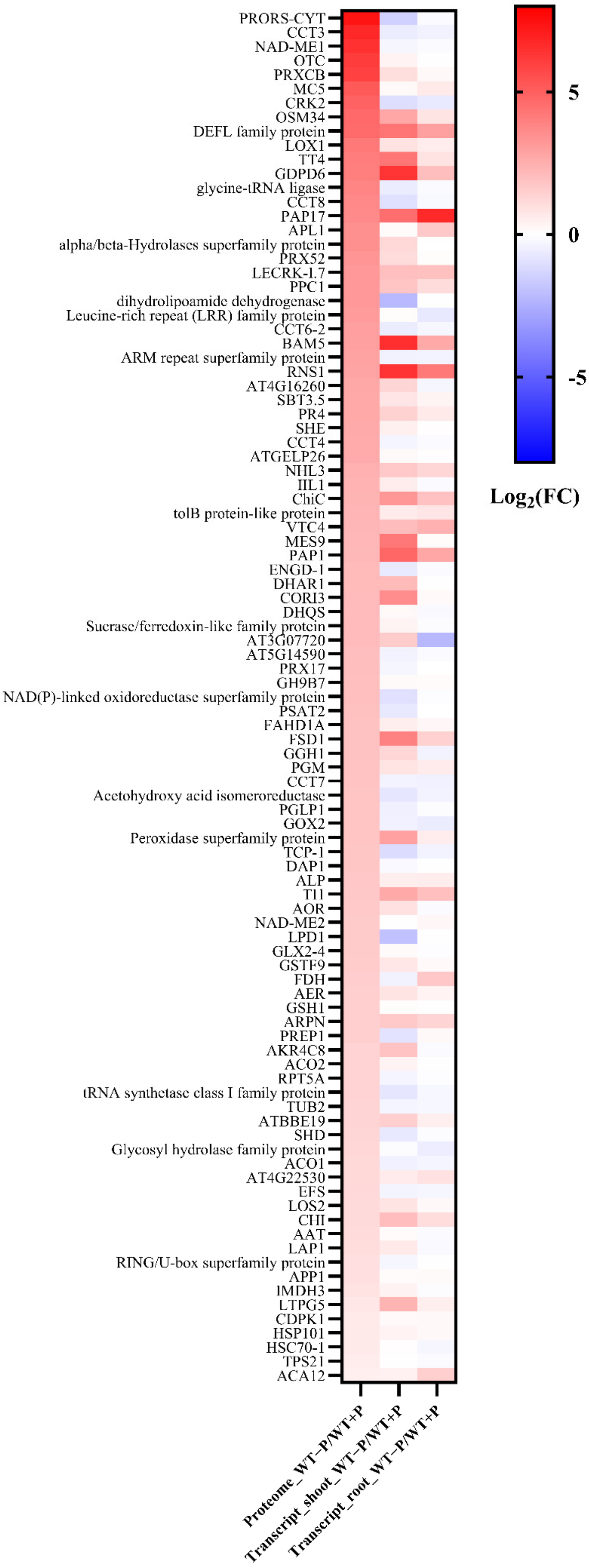
PRORS-CYT
CCT3
NAD-ME1
OTC
PRXCB
MC5
CRK2
OSM34
DEFL family protein
LOX1
TT4
GDPD6
glycine-tRNA ligase
CCT8
PAP17
APL1
alpha/beta-Hydrolases superfamily protein
PRX52
LECRK-I.7
PPC1
dihydrolipoamide dehydrogenase
Leucine-rich repeat (LRR) family protein
CCT6-2
BAM5
ARM repeat superfamily protein
RNS1
AT4G16260
SBT3.5
PR4
SHE
CCT4
ATGELP26
NHL3
IIL1
ChiC
tolB protein-like protein
VTC4
MES9
PAP1
ENGD-1
DHAR1
CORI3
DHQS
Sucrase/ferredoxin-like family protein
AT3G07720
AT5G14590
PRX17
GH9B7
NAD(P)-linked oxidoreductase superfamily protein
PSAT2
FAHD1A
FSD1
GGH1
PGM
CCT7
Acetohydroxy acid isomeroreductase
PGLP1
GOX2
Peroxidase superfamily protein
TCP-1
DAP1
ALP
TI1
AOR
NAD-ME2
LPD1
GLX2-4
GSTF9
FDH
AER
GSH1
ARPN
PREP1
AKR4C8
ACO2
RPT5A
tRNA synthetase class I family protein
TUB2
ATBBE19
SHD
Glycosyl hydrolase family protein
ACO1
AT4G22530
EFS
LOS2
CHI
AAT
LAP1
RING/U-box superfamily protein
APP1
IMDH3
LTPG5
CDPK1
HSP101
HSC70-1
TPS21
ACA12
Proteome_WT−P/WT+P
Transcript_shoot_WT−P/WT+P
Transcript_root_WT−P/WT+P
5
0
-5
Log2(FC)

Supplementary Figure S6. Integrated analyses of up-regulated secretome and transcriptomics in WT−P/WT+P.
